# Cross-Vascular Graft Reconstruction for Lung Cancer Involving the Upper Superior Vena Cava

**DOI:** 10.1016/j.atssr.2025.05.012

**Published:** 2025-06-06

**Authors:** Naru Kitade, Naoya Kitamura, Ryo Yokoyama, Toshihiro Ojima, Norimasa Miyoshi, Tomoshi Tsuchiya

**Affiliations:** 1 Department of Thoracic Surgery, Toyama University Hospital, Toyama City, Japan; 2 Department of Anesthesiology, Toyama University Hospital, Toyama City, Japan

## Abstract

We report a patient with a case of lung cancer invading the right brachiocephalic vein (BCV) and superior vena cava (SVC) that was managed with single prosthetic graft reconstruction. A 63-year-old patient with stage IIIA squamous cell carcinoma underwent right upper and middle lobectomy, rib resection, and vascular reconstruction after neoadjuvant chemoradiotherapy. The right BCV was clamped and transected, and the SVC was stapled obliquely at its bifurcation. A polytetrafluoroethylene graft was interposed from the right BCV to the right atrium, preserving SVC flow without cross-clamping. This technique minimized hemodynamic instability and reduced the risk of graft occlusion in tumors involving the upper SVC.

Several techniques have been described for replacing the superior vena cava (SVC) and brachiocephalic vein (BCV) after combined resection for malignant tumors, including SVC reconstruction alone or bilateral BCV reconstruction.^1^ However, such methods carry risks, such as severe hemodynamic alterations and neurologic complications, resulting from SVC cross-clamping.^2^ Additionally, the use of multiple prosthetic grafts increases the risk of graft occlusion secondary to reduced blood flow velocity.^3^

We present a novel approach for radical resection of non-small cell lung cancer partially invading the SVC. This approach involves stapler-assisted transection and reconstruction of the right BCV by using a single prosthetic graft. This report highlights this technique’s advantages over traditional methods.

A 72-year-old man presented with chest radiographic abnormality. Contrast-enhanced chest computed tomography revealed an 85 mm × 72 mm × 95 mm mass in the right upper and middle lobes involving the chest wall, the right BCV, and the SVC (Figure 1A). Brain magnetic resonance imaging, fluorine-18 fluorodeoxyglucose positron emission tomography, and bronchoscopy confirmed stage IIIA (cT4 N0 M0) squamous cell carcinoma of the upper and middle lobes.

**Figure 1:**
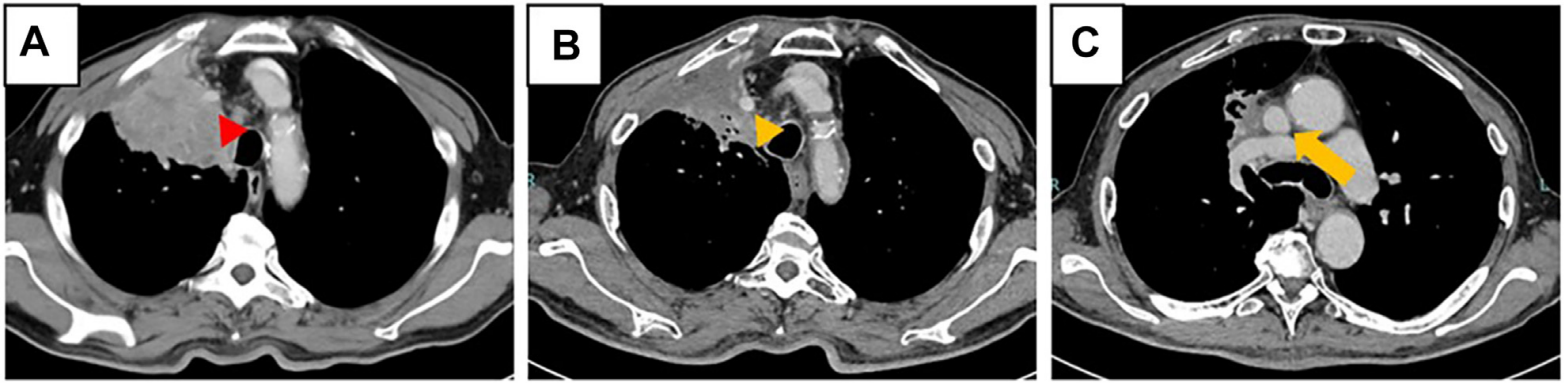
(A) Contrast-enhanced chest computed tomography before chemoradiotherapy, with the red arrowhead indicating the right brachiocephalic vein (right BCV). (B and C) Contrast-enhanced chest computed tomography after chemoradiotherapy, with (B) the orange arrowhead indicating the BCV and (C) the orange arrow indicating the superior vena cava. After chemoradiotherapy, the tumor size was reduced, and vascular invasion was mild.

Neoadjuvant chemoradiotherapy, comprising 6 courses of carboplatin and paclitaxel and 45 Gy of radiation delivered in 25 fractions, was administered. This treatment resulted in tumor shrinkage, with the disease reclassified as stage IIB (ycT3 N0 M0) and with a maximum tumor diameter of 56 mm (Figures 1B, 1C). The surgical procedure was performed 31 days after completion of neoadjuvant chemoradiotherapy.

The patient was placed in the supine position. An initial port was inserted in the right seventh intercostal space along the anterior axillary line, followed by a hemiclamshell incision and a transmanubrial approach. Intraoperatively, primary invasion of the right BCV and localized involvement of the upper SVC were confirmed (Figure 2A). Using a surgical stapler (Signia Small Diameter Reload [SDR], Medtronic), the right BCV was obliquely transected from the junction of the 2 BCVs to the upper SVC, thereby preserving venous flow in the left BCV (Figure 2B). A 5-mm margin was maintained between the tumor and the stapler line. The tumor-infiltrated segment of the right BCV was resected, and reconstruction was performed using a 10-mm ringed polytetrafluoroethylene (PTFE) graft, anastomosed between the right BCV and the right atrial appendage (RAA) with 5-0 (CV-5) PTFE sutures (Gore-Tex, W.L. Gore & Associates). The graft was placed anterior to the SVC, and it bridged the transected right BCV and the RAA without obstructing SVC flow (Figures 2C, 2D). Right upper rib resection and a middle lobectomy with 2a to 2 lymph node dissection were performed. Chest wall reconstruction was achieved using a 15 cm × 12 cm, 2-mm-thick Gore-Tex expanded PTFE patch (W. L. Gore & Associates), which was secured to the ribs with 2-0 nylon sutures. The operative time was 659 minutes, and the blood loss was 1880 g.

**Figure 2:**
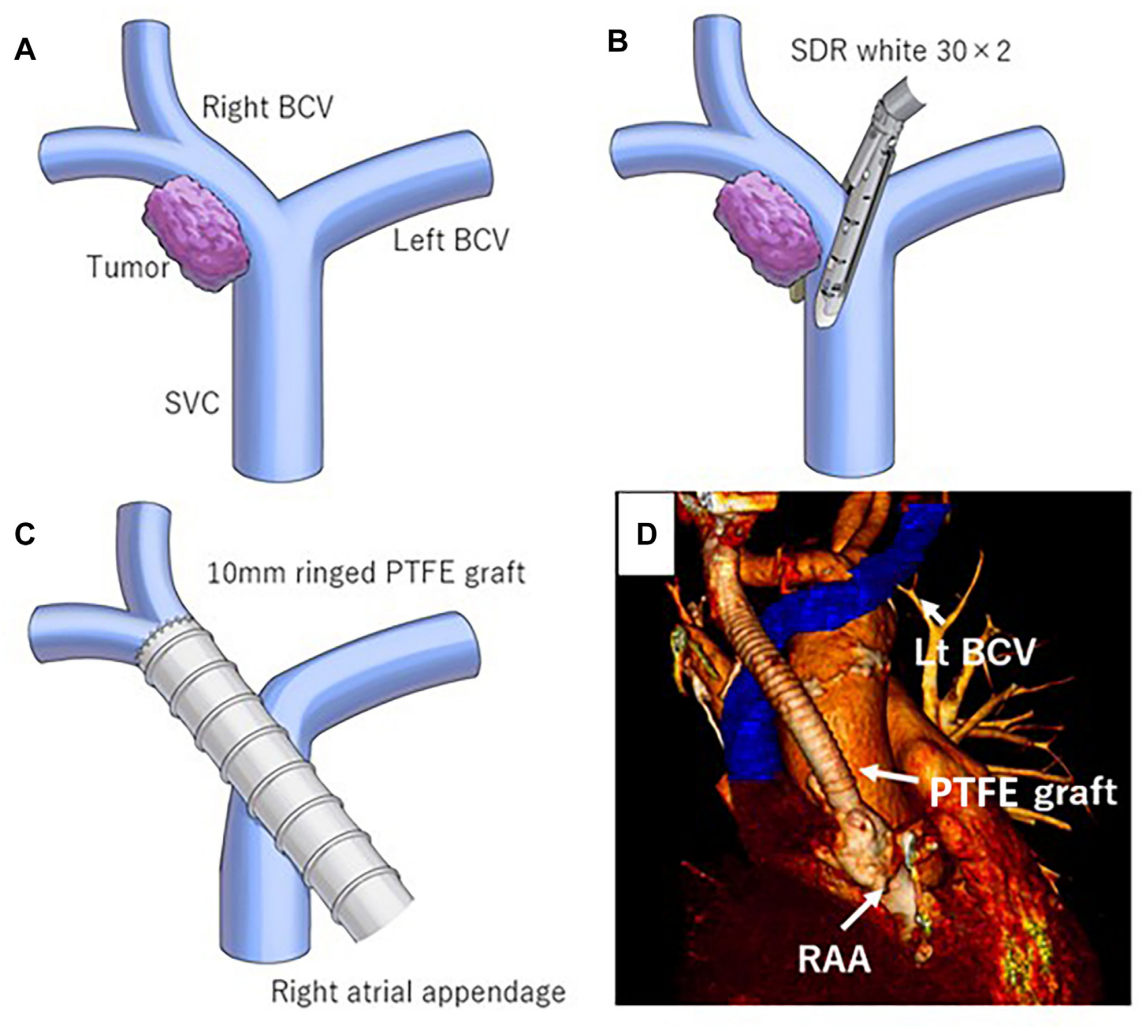
(A) The tumor was infiltrating the right brachiocephalic vein (BCV) distal to its junction with the left (Lt) BCV. (B) We dissected the right BCV using a surgical stapler. (C) Reconstruction using a 10-mm ringed polytetrafluoroethylene (PTFE) graft. Vascular reconstruction using a 10-mm ringed PTFE graft, which crossed on the left BCV to the superior vena cava. (D) The right BCV was anastomosed to the right atrial appendage (RAA). (SDR, Signia Small Diameter Reload; SVC, superior vena cava.)

Postoperatively, 5000 IU/d of intravenous heparin was administered, and oral apixaban was initiated on postoperative day 1. The postoperative course was uneventful, and the patient was discharged on day 14. The final pathologic diagnosis confirmed squamous cell carcinoma, stage IIB (pT3 N0 M0), with negative margins. Contrast-enhanced chest computed tomography performed 6 months postoperatively confirmed graft patency.

## Comment

T4 tumors with SVC invasion, once considered inoperable, may now be surgically treated after neoadjuvant chemotherapy, thus increasing the demand for prosthetic SVC replacement. A single-institution cohort study demonstrated a 5-year survival rate of 40% after prosthetic SVC replacement, a finding supporting the feasibility of this option.^4^

This case demonstrates the effectiveness of stapler-assisted transection and single graft reconstruction for primary right upper lobe lung cancer with partial invasion of the right BCV extending to the SVC. SVC cross-clamping during resection is associated with significant hypotension in approximately 30% of cases.^2^ Traditional bilateral BCV reconstruction involves connecting the right BCV to the RAA and interposing the left BCV to either the SVC or the RAA.^1^ However, this technique requires 2 prosthetic grafts, and it may increase the risk of thrombosis.^3^

The present technique avoids SVC cross-clamping and bilateral BCV replacement with 2 grafts. The SVC was transected obliquely at the BCV bifurcation while preserving the azygos vein by using a stapler. This maneuver allowed separation of the right BCV and the SVC without disrupting blood flow from the left BCV and the azygos vein into the SVC. The invaded portion of the right BCV was resected, and reconstruction was performed using a ringed PTFE graft connecting the right BCV stump to the RAA. Thinner staplers ensured better surgical margins; 2-row staplers, such as the SDR, were preferred over 3-row staplers. Although the prosthetic graft was positioned across the SVC, no thrombotic events were observed at the 6-month follow-up.

Although the patient in this case underwent neoadjuvant chemoradiotherapy, neoadjuvant immunotherapy has demonstrated improved overall survival in non-small cell lung cancer, as shown in trials such as the Neoadjuvant Study of Nivolumab Plus Ipilimumab or Nivolumab Plus Chemotherapy Versus Chemotherapy Alone in Early Stage Non-Small Cell Lung Cancer (CheckMate 816).^5^ Furthermore, neoadjuvant nivolumab administration resulted in a 90.2% downstaging rate.^6^ Given this downstaging effect, some stage III cases have been successfully treated using uniportal resection,^7^ and pneumonectomy has been avoided in favor of lobectomy in selected patients.^8^ These findings suggest that, even in patients with circumferential SVC invasion, the present approach may become feasible because of tumor shrinkage achieved through neoadjuvant immunotherapy.

One limitation is the procedure’s restricted applicability to tumors located lateral to the right BCV. Adequate dissection of adhesions at the BCV bifurcation and SVC separation sites is also required to ensure surgical feasibility. The technique is suitable for tumors that do not exhibit circumferential vascular invasion or adhesion at the resection margins. Further large-scale studies are required to validate its efficacy in reducing thrombosis risk.

In patients with primary lung cancer partially invading the SVC from the right BCV, cross-vascular reconstruction using stapler-assisted transection of the right BCV with anastomosis of a single prosthetic graft from the right BCV to the RAA may reduce the risk of graft occlusion.
